# Multiple gene expression in plants using MIDAS‐P, a versatile type II restriction‐based modular expression vector

**DOI:** 10.1002/bit.28073

**Published:** 2022-03-16

**Authors:** Elizabeth C. Pinneh, Craig J. van Dolleweerd, Kathrin Göritzer, Pascal M. W. Drake, Julian K‐C. Ma, Audrey Y‐H. Teh

**Affiliations:** ^1^ Molecular Immunology Unit, Institute for Infection and Immunity St. George's University of London London UK; ^2^ Protein Science & Engineering, Callaghan Innovation, School of Biological Sciences University of Canterbury Christchurch New Zealand

**Keywords:** anti‐HIV cocktail, Griffithsin, MIDAS‐P, modular, tandem plant expression vector, VRC01

## Abstract

MIDAS‐P is a plant expression vector with blue/white screening for iterative cloning of multiple, tandemly arranged transcription units (TUs). We have used the MIDAS‐P system to investigate the expression of up to five genes encoding three anti‐HIV proteins and the reporter gene DsRed in *Nicotiana benthamiana* plants. The anti‐HIV cocktail was made up of a broadly neutralizing monoclonal antibody (VRC01), a lectin (Griffithsin), and a single‐chain camelid nanobody (J3‐VHH). Constructs containing different combinations of 3, 4, or 5 TUs encoding different components of the anti‐HIV cocktail were assembled. Messenger RNA (mRNA) levels of the genes of interest decreased beyond two TUs. Coexpression of the RNA silencing suppressor P19 dramatically increased the overall mRNA and protein expression levels of each component. The position of individual TUs in 3 TU constructs did not affect mRNA or protein expression levels. However, their expression dropped to non‐detectable levels in constructs with four or more TUs each containing the same promoter and terminator elements, with the exception of DsRed at the first or last position in 5 TU constructs. This drop was alleviated by co‐expression of P19. In short, the MIDAS‐P system is suitable for the simultaneous expression of multiple proteins in one construct.

## Graphical abstract

Using MIDAS‐P, a modular plant expression vector, to express a cocktail of anti‐HIV molecules.

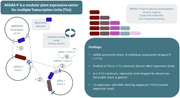

## 1 INTRODUCTION

The ability to assemble different recombinant gene modules in a combinatorial mix and match fashion is highly desirable for various applications such as the engineering of novel biological pathways, the expression of multisubunit proteins such as secretory IgA (Teh et al., 2021), interacting protein partners such as the membrane‐bound IgM/D and the Igα/Igβ heterodimer of the B‐cell receptor complex (Treanor, 2012), as well as expression of virus‐like particles for vaccine production (Marsian & Lomonossoff, 2016). Furthermore, the production of biologics that are increasingly used in combinations (see Chauhan et al., 2020 for review), where the cost of individual manufacturing lines is prohibitive to implementation, needs to be addressed.

To date, a common strategy for transient expression of multiple recombinant proteins in plants is co‐transformation with individual *Agrobacterium* strains each harboring a vector coding for a single gene of interest (Giritch et al., 2006; Roy et al., 2010). This approach can be problematic as it is not possible to ensure that the transformed plant cells simultaneously receive all of the constructs. There are a number of reports of multiple recombinant genes expressed in tandem from a single expression vector using transient expression (Sarrion‐Perdigones et al., 2013; Teh et al., 2014; van Dolleweerd et al., 2014), while reported examples for stable transformation of multiple genes in plants include the expression of up to 10 transgenes for glycan engineering by *Agrobacterium*‐mediated transformation (reviewed in Montero‐Morales & Steinkellner, 2018) and up to thirteen transgenes (Chen et al., 1998) by particle bombardment. For the insertion of multiple genes arranged in tandem in a single vector, binary bacterial artificial chromosomes (BIBAC) (Hamilton et al., 1996) and yeast artificial chromosomes (YAC) (Mullen et al., 1998) have been used. The highest number of linked genes arranged in tandem and expressed *in planta* using a single vector is 10 genes, with a single 33.6 kb T‐DNA, utilizing Cre/loxP site‐specific recombination and a transformation‐competent artificial chromosome (TAC) based vector (Lin et al., 2003). However, classical cloning of linked transgenes in one vector to create large T‐DNAs can be an overly complex process, as the finite number of available restriction enzymes becomes a limiting factor.

In recent years, a plethora of different strategies allowing the assembly of different “bioparts” such as promoters, terminators, and transcription factors have been reported (Engler et al., 2008; Knight, 2003; Rebatchouk et al., 1996; Shetty et al., 2011). More recently, a modular idempotent DNA assembly system (MIDAS) was reported (van Dolleweerd et al., 2018). This is a hierarchical cloning assembly toolkit based on the Golden Gate use of type IIS restriction enzymes to generate non‐palindromic overhangs that ligate upon addition of a ligase in a “one‐pot” reaction. This allows the assembly of genes from basic, reusable parts and the assembly of plasmids containing multiple genes. Using the MIDAS system, the group was able to successfully assemble seven genes from 21 modules in a single plasmid and demonstrate expression in *Penicillium paxilli* (van Dolleweerd et al., 2018).

We have generated a simple, non‐hierarchical version of MIDAS, named Modular Idempotent DNA Assembly System for plants (MIDAS‐P), to investigate expression of multiple genes *in planta*. The TUs are first prefabricated in entry vectors, followed by a strategy using type IIS restriction sites and alternating blue/white screening that can arrange these multiple TUs in a binary destination vector. We used MIDAS‐P to assemble plasmids designed to express a cocktail of anti‐HIV biologics as a test case. The cocktail of anti‐HIV therapeutics include (1) VRC01, a potent and broadly neutralizing antibody that has been shown to neutralize 91% of HIV‐1 isolates by targeting the CD4 binding site of the virus (Wu et al., 2010); (2) the lectin Griffithsin (GRFT), which targets the HIV envelope glycans and has subnanomolar activity against CXCR4‐and CCR5‐tropic strains of HIV‐1 (Emau et al., 2007; Mori et al., 2005); and (3) the camelid single‐chain nanobody J3‐VHH which targets the CD4 binding site of HIV and has been shown to neutralize 96% of HIV‐1 strains tested (McCoy et al., 2012). The fluorescent reporter protein DsRed was also included.

We also aimed to test the capacity of this novel assembly toolkit for the transient expression in *Nicotiana benthamiana* tobacco plants by cloning up to five genes in tandem in one vector in different permutations. We assessed what effect the number of TUs in a construct, and their relative positions, can have on the mRNA and recombinant protein expression levels of each cocktail component.

## 2 EXPERIMENTAL PROCEDURES

### 2.1 Cloning of VRC01, GRFT, DsRed, J3‐VHH, and P19

VRC01 heavy HC and LC with a human Ig gamma or kappa leader sequence (Teh et al., 2014), GRFT (Hoelscher et al., 2018), J3‐ VHH (McCoy et al., 2012) with a 6xhis‐tag followed by a C‐terminal KDEL‐tag, and the codon optimized gene from *Discosoma* sp. fluorescent protein FP583 R2G mutant (DsRed) (AF168419) with a six amino acid (SATGSA) chloroplast‐targeting N‐terminal transit peptide from potato starch granule‐bound starch synthase (GBSS) were amplified using relevant templates and primers (Table S1). The genes were domesticated to be free from NcoI, XbaI, BsaI, and BsmBI sites. The exception is the DsRed where NcoI was present. The P19 gene silencing suppressor of Tomato bushy stunt virus (ACV49953.1) was amplified from pEAQ‐HT‐DEST3 (Sainsbury et al., 2009) and the internal BsaI site was removed using Quickchange II mutagenesis kit (GGTCTC to GCTCTC) as per manufacturer's instructions (Agilent, USA). The 5ʹ and 3ʹ ends of the sequences were flanked with NcoI and XbaI restriction sites, respectively, and cloned into either pWHITE or pBLUE depending on their assembly position in the destination vector pMIDAS (Table 1). For DsRed, the forward primer had a BsaI followed by an NcoI restriction site to generate compatible cohesive ends to the NcoI site in pWHITE or pBLUE. Therefore, the DsRed PCR products were digested with BsaI and XbaI (BsaI site was removed after digestion). Ligated constructs were transformed into *E*. coli DH5α (Thermo Fisher) and plated on Luria‐Bertani (LB) agar containing 50 µg/ml kanamycin (Apollo Scientific) for plasmid storage and replication.

The entry vector pWHITE harboring the first transcription unit (TU) was ligated into the pMIDAS destination vector using BsaI. Briefly, 100 ng of entry vector were mixed with 100 ng of destination vector together with 10 units of BsaI (NEB), 400 units of T4 DNA ligase (NEB, USA) and 1x T4 DNA ligase buffer. The mixture was cycled 50 times between 37°C for 2 min and 16°C for 5 min before ending with 37°C for 5 min. The mixture was then transformed into *Escherichia coli* DH10B (Thermo Fisher) and spread onto LB selection plates containing 50 µg/ml carbenicillin (Apollo Scientific), 1 mM IPTG, and 20ug/ml X‐Gal. White colonies were selected.

In the next step, the second TU, which is cloned in the pBLUE entry vector, was assembled in the same way into the destination vector pMIDAS + TU1 using BsmBI, transformed into DH10B and plated onto selection plates. Blue colonies were selected. The third, fourth, and fifth TUs were consecutively ligated into the destination vectors using BsaI (for pWHITE TUs) or BsmBI (for pBLUE TUs). The constructs were then transformed into *Agrobacterium tumefaciens* GV3101::pMP90(RK) and plated onto Yeast‐extract mannitol (YM) medium (0.04% w/v Yeast extract, 1% w/v Mannitol, 1.7 nM NaCl, 0.8 mM MgSO4, 2.2 nM K_2_HPO_4_, pH7) with 100 µg/ml rifampicin, 50 µg/ml kanamycin, 50 µg/ml gentamycin, and 50 µg/ml carbenicillin.

### 2.2 Transient expression of VRC01, GRFT, J3‐VHH, DsRed, and P19

*A. tumefaciens* GV3101:pMP90(RK) transformed with pMIDAS containing the genes of interest were grown overnight at 28°C in LB broth supplemented with 100 µg/ml rifampicin, 50 µg/ml kanamycin, and 50 µg/ml carbenicillin (all Apollo Scientific). After removal of the medium by centrifugation, the pellet was resuspended in infiltration solution containing 0.01 mM MES (Sigma) pH 5.6, 0.01 mM MgCl_2_ (VWR International) and 0.1 mM acetosyringone (Santa Cruz Biotechnology). The final infiltration OD_600_ of the bacterial suspensions was adjusted to 1.0 for the 1 to 5 TUs experiment (Figure 2), and lowered to 0.1 for subsequent experiments to reduce leaf necrosis. Fully expanded leaves of 3.5–5‐week‐old *N. benthamiana* ΔXT/FT plants (Strasser et al., 2008) were either transformed using syringe‐mediated infiltration or vacuum infiltration as described by Kapila et al. (1997). The plants were then further grown in containment at 25°C with a 16/8‐h light/dark cycle. Leaves were harvested at 6 days postinfiltration (dpi). Plant crude extract was obtained by grinding the leaves using pestle and mortar, or 3 mm chrome steel ball bearings and a Mixer Mill MM400 (Retsch) with 3 ml 1xphosphate‐buffered saline (PBS) (2.7 mM KCl, 8 mM Na_2_HPO_4_, 137 mM NaCl, 2 mM KH_2_PO_4_) per 1 g leaf fresh weight. Total soluble protein (TSP) of the crude extract was measured at A_280_ with a Nanodrop 2000 (Thermo Fisher).

### 2.3 Western blot detection of VRC01, GRFT, and J3‐VHH from plant leaf extracts

Plant homogenates containing 30 µg of TSP were mixed with non‐reducing NuPAGE LDS sample buffer and boiled for 10 min. Proteins were separated on a 4%–12% Bis‐Tris NuPAGE gel (Life Technologies) with MOPS buffer and transferred to a nitro‐cellulose membrane for immunoblotting. The membrane was blocked with 5% w/v non‐fat dried milk (NFDM) in Tris‐buffered saline supplemented with 0.1% v/v Tween20 (TBS‐T) before being probed with either peroxidase‐conjugated polyclonal sheep anti‐human kappa LC antibody (1 in 10,000), peroxidase‐conjugated sheep anti‐human IgG gamma chain (1 in 5000), peroxidase‐conjugated monoclonal anti‐polyhistidine antibody (1 in 2500; all from Sigma) or rabbit anti‐GRFT (1 in 2000; gift from Barry O'Keefe). Anti‐GRFT blots were subsequently incubated with peroxidase‐conjugated sheep anti‐rabbit IgG antiserum (1 in 2000; Sigma). Protein detection was done using the ECL Prime system (Thermo Fisher) and were visualized using G:Box F3 (Syngene, UK) or Amersham Hyperfilm ECL (GE Healthcare).

### 2.4 DsRed fluorescence measurements

Homogenized crude plant extracts were titrated 1:1 seven times on a 96‐well ELISA plate (50 μl volume; Corning) in triplicate. Purified plant‐made DsRed protein (gift from Fraunhofer IME) was used as a standard and/or positive control. Leaves infiltrated with *Agrobacterium* harboring pMIDAS empty vector were used as a negative control. Readings were carried out at 590 nm using the Infinite F200 Pro plate reader (TECAN). Each point was measured in triplicate.

### 2.5 Genomic DNA extractions and insert amplification

Genomic DNA was extracted from leaf samples using DNeasy plant mini kit (QIAGEN) according to the manufacturer's instructions. PCR was carried out using HF Phusion master mix (NEB) with PCR cycle according to manufacturer guidelines using gene‐specific primers detailed in Table S1. Negative controls were leaves infiltrated with pMIDAS alone and positive controls were leaves infiltrated with pMIDAS harboring VRC01 HC + LC, GRFT, J3‐VHH, or DsRed depending on the experiment.

### 2.6 Taqman qPCR

RNA was extracted using RNeasy plant mini kit according to the manufacturer's instructions and treated with DNase using RNase free DNase set (all QIAGEN). Following DNase treatment, RNA was quantified with a NanoDrop 2000 (Thermo Scientific) and 200 ng of RNA were used to synthesis cDNA using the LunaScript RT supermix kit (NEB). The synthesized cDNA was diluted 1:50 and 5 µl were used for a qPCR reaction with the addition of 15 µl mixture of GoTaq probe qPCR master mix (Promega), target specific forward primers (250 nM), reverse primers (250 nM), and 6FAM/BHQ1 internal Taqman probes (900 nM). The ribosomal protein L25 was used as a reference gene and qPCR thermocycle protocol was carried out according to qPCR master mix manufacturer's instructions; reactions were performed using the CFX‐connect real time PCR detection system (Biorad). All primers were designed using Primerplus and were purchased from Sigma. Primers used for Taqman qPCR are summarized in Table S2.

### 2.7 ELISA

Sheep anti‐human IgG antiserum (5 µg/ml; AU004, The Binding Site; for VRC01) or 15 µg/ml UG37gp140 (ARP0698, CFAR; for GRFT and J3‐VHH) diluted in PBS were used to coat Nunc Maxisorp ELISA plates (Thermo Fisher) for 2 h at 37°C, followed by blocking for 1 h with 5% w/v NFDM in PBS + 1% v/v Tween20 (PBS‐T). Plates were then incubated with crude plant extract at 37°C for 2 h.

For detection, incubation was with either 1 in 1000 peroxidase‐conjugated sheep anti‐human IgG gamma chain antiserum (for VRC01), 1 in 1000 rabbit anti‐GRFT antiserum (for GRFT) or 1 in 2000 peroxidase‐conjugated monoclonal antipolyhistidine antibody (for J3‐VHH) for 1 h. Anti‐GRFT ELISAs were followed by further incubation with 1 in 2000 peroxidase‐conjugated sheep anti‐rabbit IgG. Bound peroxidase‐conjugated antibodies were detected using 3,3′,5,5′‐tetramethylbenzidine (TMB) substrate solution (Sigma). The color reaction was stopped by addition of 2 M sulfuric acid and the absorbance was measured at 450 nm using the Infinite F200 Pro plate reader (TECAN). Titrations of IgG1 kappa LC from human myeloma (Sigma), plant‐produced GRFT (gift from Evangelia Vamvaka and Paul Christou) or plant‐made J3‐VHH at known concentrations were used as standards to determine protein concentration.

### 2.8 Statistical analysis

Normality of all data was tested and null hypothesis rejected if *p* < 0.05 using the Shapiro–Wilk test; if found to be normal, a one‐way ANOVA test was carried out. If data was significantly skewed, a non‐parametric Kruskal–Wallis test was used for data analysis. The homogeneity of variance was also tested for each sample data set using the Levene test and the null hypothesis was rejected if *p* < 0.05 resulting in the analysis of a data set either with the Brown–Forsythe and Welch correction or ordinary one‐way ANOVA test depending on Levene test outcome. Post hoc analysis of the different sample groups was carried out either using the Dunn Bonferroni or the Tamhane T2 post hoc multiple comparison test depending on the outcome of the Levene test. All graphs were drawn and analyzed using the GraphPad Prism 8 software (GraphPad, USA).

## 3 RESULTS

### 3.1 Design of MIDAS‐P entry and destination vectors

**Figure 1:**
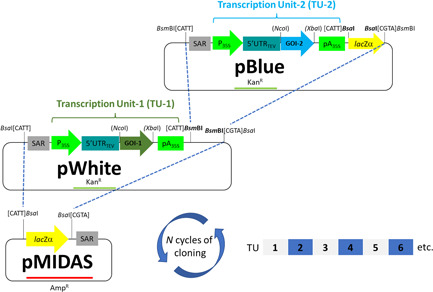
Schematic representation of the MIDAS‐P assembly system for plant expression. The system consists of two entry vectors, pWHITE and pBLUE, for cloning genes of interest and alternate sub‐cloning in the binary destination (expression) vector pMIDAS. The first transcriptional unit is constructed in pWHITE and transferred into pMIDAS using the type IIS restriction enzyme BsaI. A second transcriptional unit in pBLUE can subsequently be transferred into pMIDAS using BsmBI. Further TUs can be added by alternating transfer from pWHITE and pBLUE. The inclusion of *lacZα* in pMIDAS and pBLUE allows blue/white screening at each stage. The destination vector pMIDAS also has right and left T‐DNA borders for *Agrobacterium*‐mediated plant transformation. GOI, gene of interest; P, promoter; pA, terminator and polyA signals; SAR, scaffold attachment region; UTR, untranslated region

The MIDAS‐P assembly system for expression of multiple genes in plants comprises two entry vectors, pWHITE and pBLUE, which contain transcription units (TUs) based on the pTRAk system (Sack, 2007), and a destination expression vector, pMIDAS for accepting the TUs containing the genes of interest from the entry vectors (Figure 1).

The entry vectors pWHITE and pBLUE each contain an identical cassette which comprises the Scaffold Attachment Region (SAR) of the tobacco Rb7 gene, a cauliflower mosaic virus (CaMV) 35S promoter with duplicated enhancer, the 5ʹ untranslated region (UTR) of tobacco etch virus (TEV), gene of interest (GOI) cloning sites (NcoI/XbaI) and a CaMV 35S polyadenylation site/terminator (Figure 1). pBLUE has an additional *lacZ* gene for blue/white selection during the cloning process. The pWHITE and pBLUE vectors further differ in their type II restriction sites used for transfer of the TU into the multigene cassette assembly in the destination vector. In pWHITE, the TU is flanked by BsaI; in pBLUE, the TU is flanked by BsmBI. The GOI is cloned in pWHITE or pBLUE based simply on the order in which the GOIs are assembled into the destination vector ‐ pWHITE is used for genes going into odd‐numbered positions in the final multi‐gene cassette, while pBLUE is used for even‐number positions (Figure 1).

The assembly of the multiple genes in the destination vector pMIDAS crucially depends on the configuration of BsaI and BsmBI type IIS restriction sites in pWHITE and pBLUE, respectively. TUs assembled in pWHITE can be used for cloning into destination vectors using a BsaI‐mediated one‐pot Golden Gate assembly reaction, which introduces BsmBI sites that are used for addition of the next TU. In turn, cloning from pBLUE introduces new BsaI sites, allowing cloning again from pWHITE. This cycle of cloning can be repeated indefinitely, and each plasmid generated by cloning a TU into the multigene construct becomes the destination vector for the next cycle of TU addition. Following each cloning cycle, positive clones can be identified by blue or white colony screening.

### 3.2 Co‐expression of multiple target proteins

**Figure 2:**
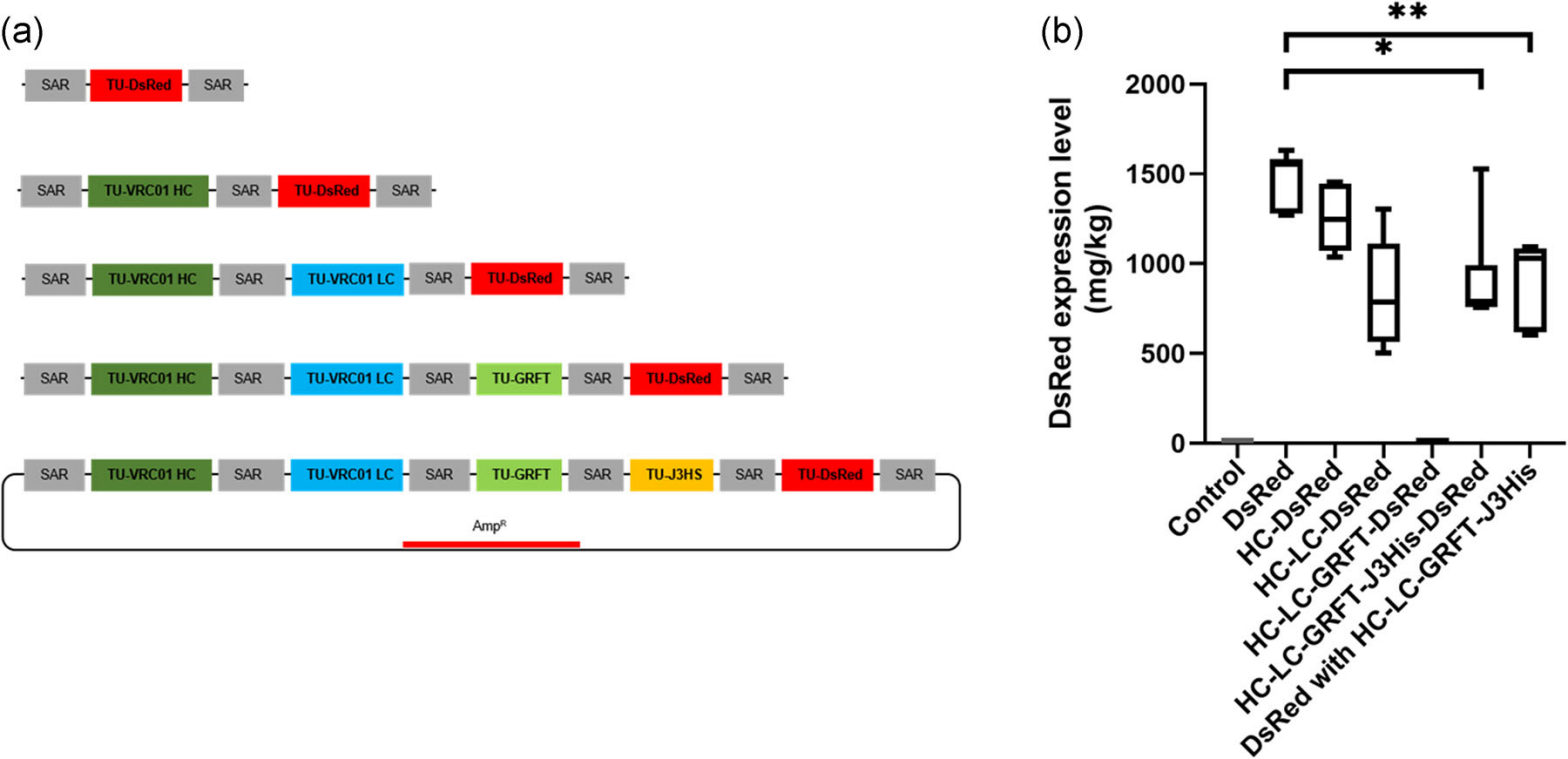
DsRed expression level with increasing number of TUs. (a) The arrangement of the individual transcription units in tandem in pMIDAS. DsRed (red box) was cloned into the last position in constructs harboring 2–5 TUs. The other TUs can include VRC01 heavy chain (HC; dark green), VRC01 light chain (LC; blue), Griffithsin (GRFT; light green) and 6x‐His tagged J3‐VHH (J3HS; yellow). (b) DsRed expression levels in leaves infiltrated with 1 TU (DsRed), 2 TUs (HC‐DsRed), 3 TUs (HC‐LC‐DsRed), 4 TUs (HC‐LC‐GRFT‐DsRed), or 5 TUs (HC‐LC‐GRFT‐J3His‐DsRed), as well as leaves co‐infiltrated with 4 TUs and DsRed as separate constructs (DsRed with HC‐LC‐GRFT‐J3His), quantified at 6 dpi. Leaves infiltrated with pMIDAS only were used as a negative control (Control). Box plot for DsRed expression levels represent the mean, minimum, and maximum of six biological repeats. Data were analyzed using Brown–Forsythe and Welch ANOVA tests with Tamhane T2 multiple comparison test (*p < 0.033 and **p < 0.002). ANOVA, analysis of variance; TUs, transcription units

First, we assessed MIDAS‐P by expressing the fluorescent reporter protein DsRed (Matz et al., 1999) as the last TU in constructs containing 1 TU (DsRed only) to 5 TUs (Figure 2A, Table 1) in *N. benthamiana*. The other TUs were populated by different anti‐HIV compounds including VRC01 light chain (LC), VRC01 heavy chain (HC) (Teh et al., 2014; Wu et al., 2010), GRFT (Emau et al., 2007; Mori et al., 2005; Vamvaka et al., 2016), and J3‐VHH including a 6xhis‐tag (J3His; McCoy et al., 2012). DsRed was also coexpressed as a separate construct together with the 4 TU construct (4TU + DsRed). The plasmids in the *Agrobacterium* were all intact before transformation into the plants, and there were no detectable recombination‐mediated rearrangements or deletions (Figure S1).

**Table 1:** **Constructs used in experiments and position of each gene of interest in the constructs**. TUs 1, 3, and 5 were cloned into pWHITE entry vectors (W) before ligated into the destination vectors; TUs 2 and 4 were cloned into pBLUE entry vectors (B). Genes of interest were VRC01 heavy chain (HC), VRC01 light chain (LC), Griffithsin (GRFT), J3‐VHH with 6xHis tag (J3His) and DsRed

Constructs	TU1 (W)	TU2 (B)	TU3 (W)	TU4 (B)	TU5 (W)
DsRed	DsRed				
HC‐DsRed	VRC01 HC	DsRed			
HC‐LC‐DsRed	VRC01 HC	VRC01 LC	DsRed		
HC‐LC‐GRFT‐DsRed	VRC01 HC	VRC01 LC	GRFT	DsRed	
HC‐LC‐GRFT‐J3His‐DsRed	VRC01 HC	VRC01 LC	GRFT	J3His	DsRed
DsRed‐J3His‐GRFT‐LC‐HC	DsRed	J3His	GRFT	VRC01 LC	VRC01 HC
GRFT‐LC‐DsRed‐J3His‐HC	GRFT	VRC01 LC	DsRed	J3His	VRC01 HC
HC‐LC‐GRFT‐J3His‐DsRed	VRC01 HC	VRC01 LC	GRFT	J3His	DsRed
GRFT‐LC‐HC	GRFT	VRC01 LC	VRC01 HC		
LC‐GRFT‐HC	VRC01 LC	GRFT	VRC01 HC		
HC‐LC‐GRFT	VRC01 HC	VRC01 LC	GRFT		
VRC01	VRC01 HC	VRC01 LC			
GRFT	GRFT				
DsRed	DsRed				
J3His	J3His				

At 6 dpi, there were no significant differences in DsRed expression level for 1‐ and 2 TU constructs (Figure 2b). There was a slight drop in the mean expression level when DsRed was expressed as a 3 TU construct but this was not statistically significant. When expressed as a 4 TU construct, DsRed expression dropped to barely detectable levels. Surprisingly, when expressed as a 5 TU construct, DsRed expression was unexpectedly restored to levels comparable to when DsRed was expressed as a 3 TU construct or when co‐expressed as a separate construct together with a 4 TU construct containing VRC01 HC and LC, GRFT, and J3‐VHH (Figure 2b).

**Figure 3:**
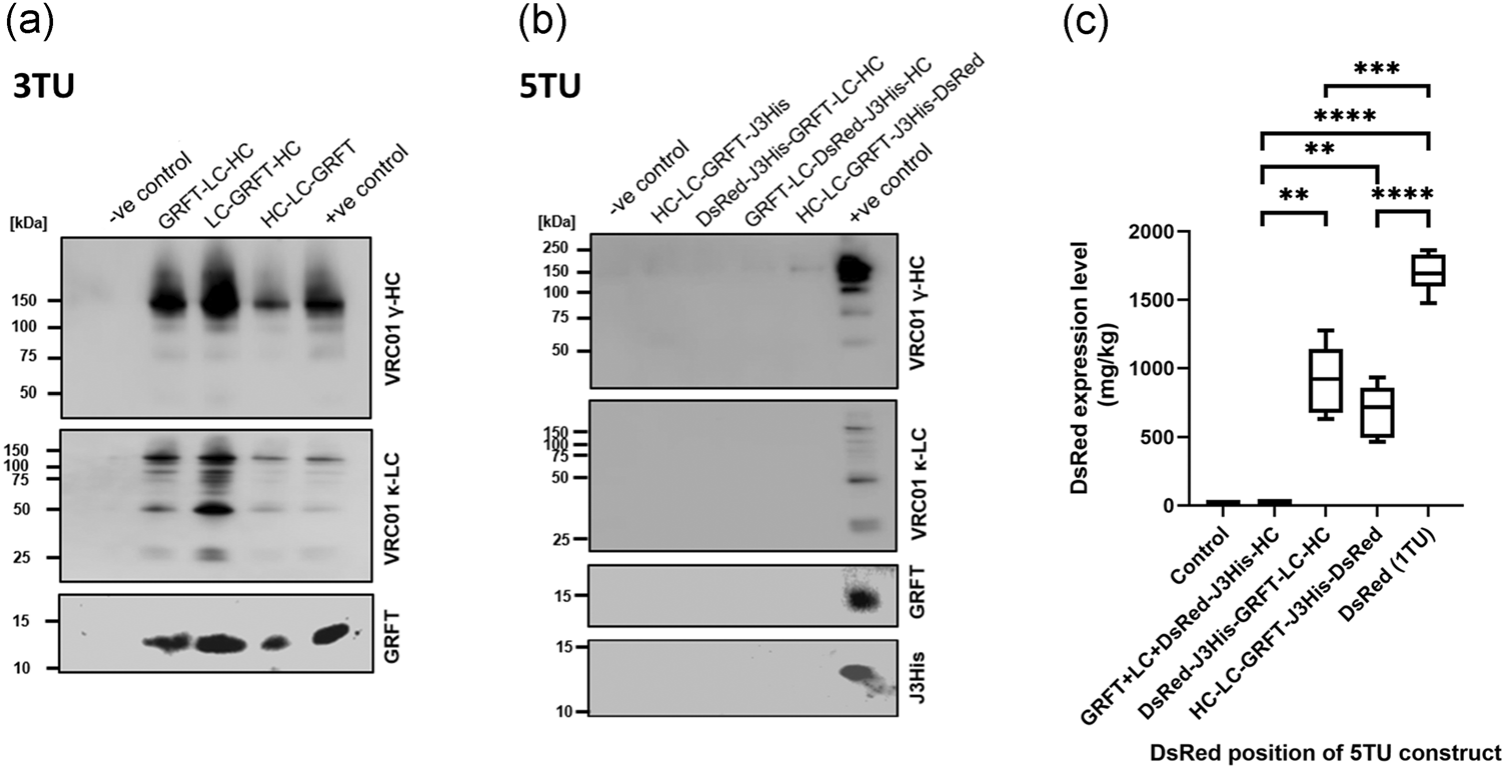
Positional effects with TU permutations on protein expression of MIDAS‐P constructs. Representative western blots of extracts from leaves infiltrated with different constructs harboring permutations of 3 TUs (a) and of 4–5 TUs (b) at 6 dpi. HC‐LC‐GRFT‐J3His is shown as a representative example of 4 TU constructs. Leaves infiltrated with pMIDAS were used as negative controls (−ve control). Positive controls (+ve control) were leaves infiltrated with either a 2 TU construct containing VRC01 HC + LC, or a 1 TU construct containing GRFT or 6x histidine tagged J3‐VHH (J3His). (c) DsRed expression levels in 4 or 5 TU constructs. Leaves infiltrated with pMIDAS only were used as negative controls (Control). Expression levels were quantified using a DsRed standard and box plots represent the mean, minimum and maximum of six biological repeats. Data were analyzed using Brown‐Forsythe and Welch ANOVA tests with Tamhane T2 multiple comparison test (***p < 0.001 and ****p < 0.0001). ANOVA, analysis of variance; TUs, transcription units

To investigate if recombinant protein expression is affected by the position of the TU in the destination vector, permutations of the constructs harboring 3–5 TUs were generated. When VRC01 HC, LC and GRFT were expressed as 3 TU constructs, similar expression levels of each recombinant protein were achieved regardless of their position in the multigene assembly (Figure S2). However, accumulation was at a significantly lower levels compared to when GRFT was expressed alone (Figure S2). When the multigene constructs contained 4 or 5 TUs, no expression of VRC01 LC or HC, GRFT or J3‐VHH could be detected by western blot (Figure 3b) with the exception of DsRed when positioned first or last in a 5 TU construct (Figure 3c).

The presence of constructs harboring 4 or 5 TUs in the plant leaves 6 days after *Agrobacterium* infiltration was confirmed, even though protein expression was not detected. DNA was isolated from infiltrated leaves and the target sequences were amplified with gene‐specific primers (Table S1). Leaves infiltrated with vector only and purified plasmids containing the single GOI were used as negative and positive controls, respectively. All genes of interest in the multi‐gene constructs were detected in DNA isolated from infiltrated leaves (Figure S3). However, we were not able to distinguish between T‐DNA that were transferred into plant cells and the plasmids still contained in the *Agrobacterium* using this method.

### 3.3 Loss of expression is due to decreased intact mRNA

With the exception of DsRed at the first or last position of a 5 TU construct, we showed that no recombinant protein expression was detected in leaves infiltrated with constructs with 4 or more TUs, regardless of their relative positions within the destination vector (Figure 3b). In the next step, we used qPCR to quantify mRNA levels of each target gene when expressed alone, or in different combinations of 3, 4, and 5 TUs, assembled by permutations in the destination vector.

**Figure 4:**
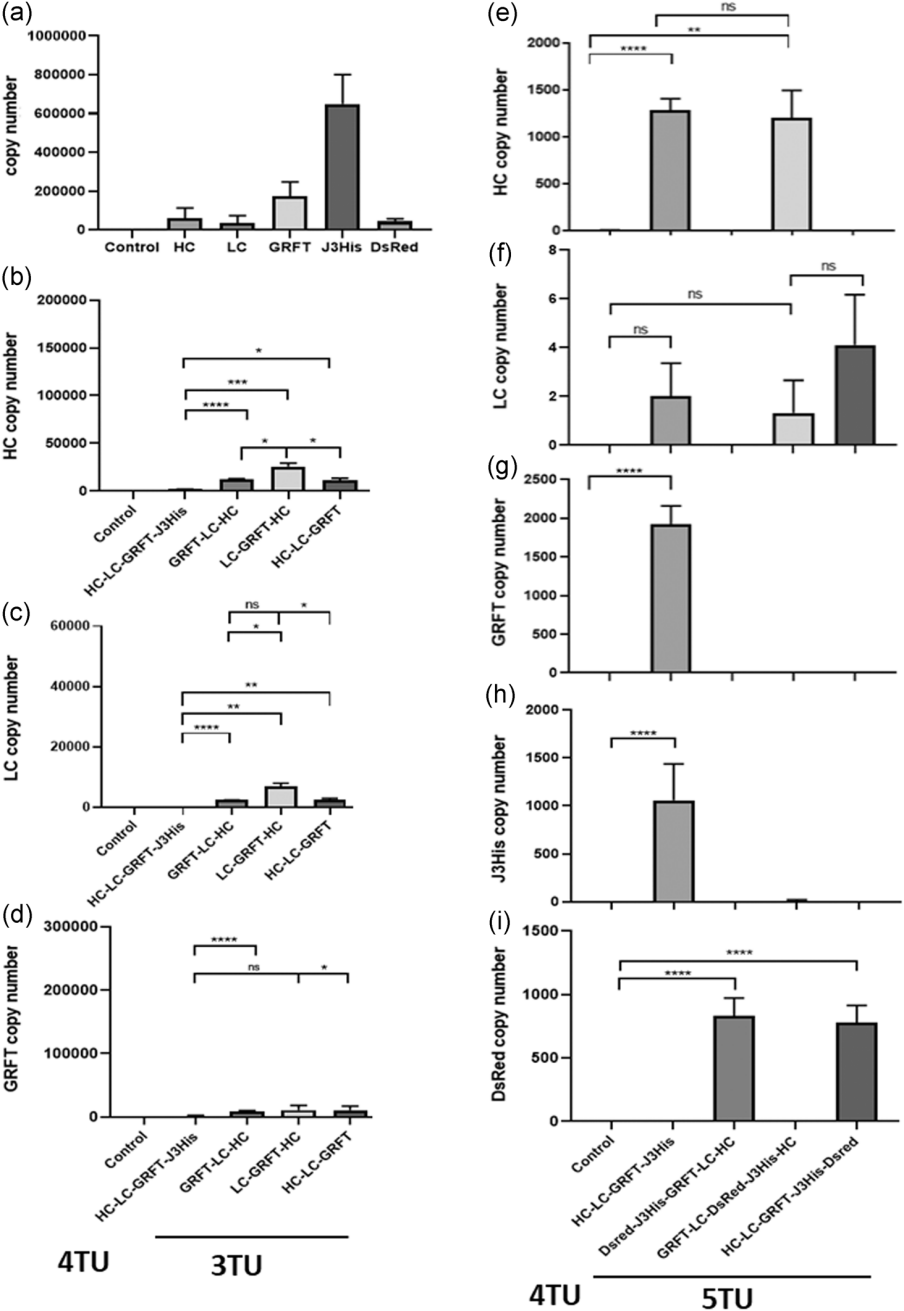
Quantification of mRNA levels of *Nicotiana benthamiana* leaves infiltrated with MIDAS‐P constructs harboring different combinations of transcription units. (a) Positive controls showing mRNA levels in leaves infiltrated with MIDAS‐P constructs carrying two TUs for VRC01 heavy chain (HC) and light chain (LC), or a single TU for expression of Griffithsin (GRFT), 6xHis‐tagged J3‐VHH (J3His) or DsRed (b–d) VRC01 HC (b), LC (c) and GRFT (d) mRNA levels in leaves infiltrated with MIDAS‐P constructs with different permutations of 3 TUs expressing VRC01 HC, LC, and GRFT; or with 4 TUs (additional TU expressing J3His), compared with the respective positive controls on Figure (a) (J3His data not shown). Data for (b), (c), and (d) were analyzed using Brown‐Forsythe and Welch ANOVA tests followed by Tamhane T2 multiple comparison test (****p < 0.0001; **p < 0.01, *p < 0.05). (e–i) VRC01 HC (e), LC (f), GRFT (g), J3His (h), and DsRed (i) mRNA levels in leaves infiltrated with MIDAS‐P constructs with different permutations of 5 TUs expressing the 5 genes and a repeat of the 4 TU construct from (b–d), compared with the respective positive controls on Figure (a). Please refer to Figure 4i for x‐axis labels of e–h. Data for (e), (g), and (i) were analyzed using Brown‐Forsythe and Welch ANOVA tests with Tamhane T2 multiple comparison test; (f) and (h) were analyzed using Dunn Bonferroni multiple comparison test (LC: Kruskal–Wallis test statistic = 6.7; J3His vs. HC‐LC‐GRFT‐J3His *p* < 0.0001, Kruskal–Wallis test statistic = 40.2). Leaves from plants infiltrated with infiltration solution served as negative control (“Control”). mRNA levels were determined with qPCR using the primers listed in Table S2. All data represent the mean of three biological repeats ± *SD*. ANOVA, analysis of variance; mRNA, messenger RNA; TUs, transcription units; *SD*, standard deviation

mRNA levels of each target gene were significantly higher in leaves infiltrated with constructs harboring only 1 TU (or 2 TUs for VRC01; Figure 4a) compared with 3‐, 4‐, and 5 TU constructs (Figure 4b–i). Within permutations of 3 TU constructs, we observed a significant decrease in mRNA levels of VRC01 LC, HC, and GRFT (Figure 4b–d) compared to mRNA levels of single TU (or 2 TU in the case of VRC01) constructs (Figure 4a), regardless of their relative positions in the construct. This effect was particularly pronounced with a construct that contained J3‐VHH as an additional 4th TU (HC‐LC‐GRFT‐J3His in Figure 4b–d). For the 5 TU permutations, the mRNA levels of each GOI were either similar or significantly lower to their counterparts in the 4 TU (HC‐LC‐GRFT‐J3His) group (Figure 4e–h). The exception was DsRed when expressed as the first or last TU (Figure 4i). Thus, with the exception of DsRed in 5 TU constructs, there was no indication that position of a TU within the multi‐gene assembly can predict mRNA levels.

### 3.4 The silencing suppressor P19 can rescue protein expression

We showed that mRNA levels of the genes of interest are substantially reduced when 3 or more TUs are assembled in the multi‐gene vector regardless of TU permutation. However, this experiment did not show whether reduced mRNA levels were due to the mRNA not being transcribed or due to post‐transcription gene silencing. Therefore, the RNA silencing suppressor P19 (Voinnet et al., 2003) under control of the CaMV 35S promotor in pMIDAS was co‐infiltrated with the multigene construct harboring 3 TUs for the expression of GRFT, VRC01 LC, and HC.

**Figure 5:**
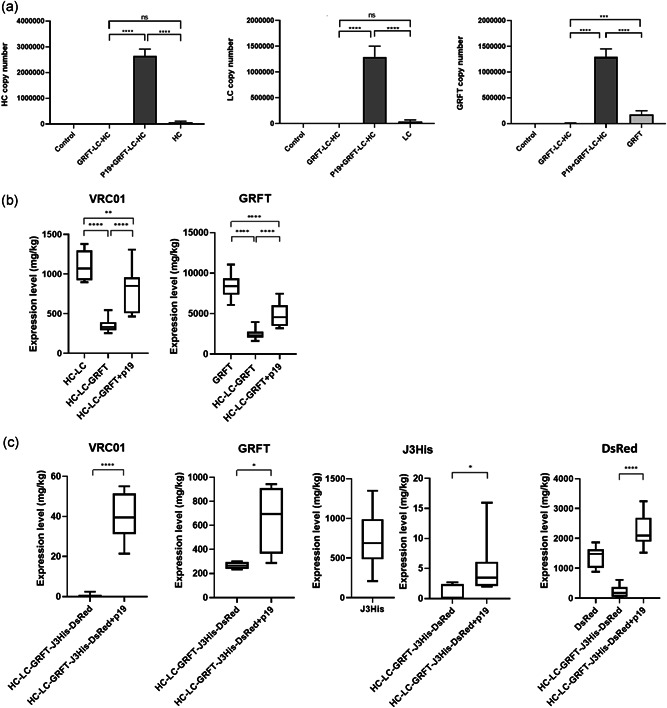
Coexpression of P19 with constructs harboring 3 or 5 TUs. (a) mRNA levels of leaves co‐infiltrated with the pMIDAS construct with 3 TUs for expression of the VRC01 heavy chain (HC), light chain (LC), and Griffithsin (GRFT) with pMIDAS‐P19 were determined by qPCR using the primers listed in Table S2. Data represent the mean of *n* = 3 biological repeats done in triplicates ± *SD*. Data were analyzed using Brown–Forsythe and Welch ANOVA tests followed by Tamhane T2 multiple comparison test (***p < 0.001 and ****p < 0.0001). (b) Protein expression levels of VRC01 and GRFT, 6x His‐tagged J3‐VHH (J3His) and DsRed expressed using 3 TU and (c) 5 TU constructs with or without P19 co‐expression. VRC01, GRFT, J3‐VHH, and DsRed expression levels were quantified by ELISA using a human IgGk standard (Sigma‐Aldrich), GRFT standard (gift from Evangelia Vamvaka and Paul Christou), plant‐made J3‐VHH and DsRed standard (gift from Johannes Buyel) respectively. Box plot for expression levels represent the median, minimum, and maximum of three biological repeats. ANOVA, analysis of variance; ELISA, enzyme‐linked immunosorbent assay; mRNA, messenger RNA; TUs, transcription units; *SD*, standard deviation

*p19* mRNA was detected when the pMIDAS‐P19 construct was expressed on its own, and co‐expressed with VRC01 in separate or the same constructs (Figure S4). Co‐infiltration of *p19* with the 3 TU construct as separate vectors caused a significant increase in (*p* < 0.0001) mRNA levels of HC, LC, and GRFT (Figure 5a). This translated to a 2–3‐fold expression level increase for both VRC01 and GRFT (Figure 5b). When *p19* was expressed as the first TU in the same vector as VRC01 (HC‐LC; Figure S5a), there were no significant differences in VRC01 expression level between P19‐HC‐LC and HC‐LC‐GRFT. However, the leaves were comparatively necrotic suggesting a hypersensitive response (HR). When P19 was expressed as the third TU, there were significant differences in VRC01 expression levels between HC‐LC‐P19 and HC‐LC‐GRFT, so much so it approached levels of the 2TU VRC01 construct (Figure S5a). On the other hand, when *p19* was expressed as the fourth TU together with VRC01 and GRFT, there was only a slight increase in expression level (9 ± 6 mg/kg compared with no expression in VRC01‐GRFT‐J3; Figure S5b).

We also investigated whether P19 co‐expression could improve the yield of VRC01, GRFT and J3‐VHH expressed as 5 TU constructs, which were at levels undetectable by Western blot (Figure 3). P19 did increase the yield of all the components of the 5 TU construct (Figure 5c). This provides evidence for the impact of post‐transcriptional gene silencing (PTGS) in constructs with 3 or more TUs, which P19 can suppress. However, VRC01 and GRFT expression from a 5 TU construct with P19 were not as high as from a 3 TU construct without P19 co‐expression (Figure 5b). For example, VRC01 expression only reached approximately 0.04 g/kg. The exception to this seemed to be DsRed, where expression levels in a 5 TU construct were higher compared with DsRed expressed in a 1 TU construct (Figure 5c).

## 4 DISCUSSION

In this study, we have created MIDAS‐P, a DNA assembly system with blue and white bacterial colony screening, for quick and easy cloning of multiple, tandemly arranged genes for expression in plants. Expression of each gene is under control of the 35S promoter. This system is made up of entry vectors pWHITE and pBLUE containing TUs which are designed for high level expression in plants. In this simple configuration, MIDAS‐P can achieve multigene assembly using only two entry vectors, which determine the order in which each TU is added to a growing multigene construct in the destination vector pMIDAS. This allows the addition of components iteratively into a single construct indefinitely (in theory), rather than having to clone long and complex constructs using different restriction enzymes. The use of the entry vectors also allows the flexibility to make multiple constructs with different configurations at the same time. For example, introducing a third TU to an existing construct that already expresses two proteins, or making constructs with permutations of the same genes of interest. Furthermore, the possibility of blue and white selection in each cycle of cloning ensures a fast and straightforward screening process.

MIDAS‐P has previously been used to express two genes of interest in tandem (Moore et al., 2021; Teh et al., 2021). In the current work, we were interested in using this system to generate multi‐gene constructs for the expression of a cocktail of anti‐HIV products, including the broadly neutralizing antibody VRC01, the lectin GRFT, and the camelid nanobody J3‐VHH. HIV is a good example of a disease target for which low‐cost cocktails of biologics (e.g., for therapy, prophylaxis, or vaccination) are likely to be needed, due to the likelihood of viral escape. We deliberately selected anti‐HIV biologics of different classes (an antibody, a lectin, and a nanobody) that were unlikely to interact with each other negatively *in planta*. Vamvaka et al. (2018) have shown that extracts of transgenic rice expressing three anti‐HIV products (2G12 antibody, GRFT, and another lectin Cyanovirin‐N) using separate constructs had synergistic HIV‐1 neutralization capabilities.

When testing the capabilities of the MIDAS‐P system, we found a tendency for reduced protein expression levels for individual proteins as the number of TUs increased. When VRC01 HC, LC, and GRFT were expressed as 3 TU constructs, mRNA levels and the corresponding protein expression decreased, although protein expression levels of up to approximately 2.5 g/kg for GRFT and approximately 0.4 g/kg for VRC01 were still achieved. Although position‐dependent differences were observed in mRNA expression, this did not impact VRC01 or GRFT expression levels. Diamos et al. (2020) reported that in a Bean Yellow Dwarf Virus (BeYDV)‐based replicating vector system, no difference in fluorescent protein accumulation levels was observed, even though there was reduced expression of the larger replicon compared to the smaller ones “split” by viral genetic elements. This is most probably due to the complex relationship between mRNA and protein expression levels (reviewed in Liu et al., 2016; McManus et al., 2015). Protein expression levels do not rely on mRNA concentration alone, and factors that can have an impact include regulation of translation rate by small RNAs (discussed below), mRNA competition for free ribosomes (Chu et al., 2011), as well as the regulation of protein concentration independent of transcript concentrations by the ubiquitin‐proteasome pathway or autophagy (Balchin et al., 2016). During state transition (e.g., cell differentiation), correlation between mRNA and protein expression levels can also be affected due to delayed synthesis between mRNA and protein (Jovanovic et al., 2015; Lee et al., 2011).

When DsRed was expressed as the fourth TU, there was no detectable DsRed expression. This was also observed when J3‐VHH was used instead of DsRed (see 4TU of Figure 3b e.g.), even though the expression vector was still detected in the leaves (Figure S3). We hypothesized that this effect might be partially caused by RNA‐mediated gene silencing (see Guo et al., 2016 for review), in particular, PTGS and/or translational repression of homologous mRNAs (Vaucheret et al., 2001; Vaucheret, 2006; Vazquez et al., 2004).

In plants expressing transgenes, previous studies have reported an over‐abundant transcription of aberrant mRNAs that lack a 5ʹ cap (Gazzani et al., 2004) or a poly‐A tail (Luo & Chen, 2007) can trigger PTGS. PTGS suppressor P19 can reduce PTGS by removing dsRNAs generated from aberrant mRNA (Silhavy et al., 2002). Here, we have shown that co‐infiltration of *p19* separately with a 3 or 5 TU construct significantly increased mRNA levels of the genes of interest, accompanied by up to 40‐fold higher recombinant protein yields. Although co‐infiltrating *p19* separately did not restore expression back to the levels observed for 1 or 2 TU constructs, co‐expression as the third TU in the same construct restored VRC01 expression to 2 TU levels. This might be due to the close proximity of P19 in the cell as it was delivered by the same vector. When P19 was co‐expressed as the first TU, no increase in protein expression was observed. This was accompanied by mild HR most likely triggered by high levels of P19 (Garabagi et al., 2012; Siddiqui et al., 2008). This might contribute to protein loss.

The *p19* co‐expression experiments confirmed that PTGS was partly contributing to the drop in mRNA levels and protein expression. High transgene expression driven by the strength of the promoter has been previously reported to trigger silencing (Que et al., 1997). In this study, we have used a CAMV 35S promoter containing a duplicated transcription enhancer, with the aim of increasing transcription activity compared to the native CAMV 35S promoter (Kay et al., 1987). This may have contributed to the production of aberrant mRNA triggering PTGS. Furthermore, all TUs contain the same promoters and enhancers. The transcription enhancer also has a 250 bp transcription activating sequence upstream of the TATA element duplicated in tandem. If siRNAs targeting the promoter or enhancer elements were generated as a result of aberrant RNA processing, this might have contributed to siRNA‐mediated TGS which targets repetitive loci for RNA‐directed DNA methylation.

Sijen et al. (2001) had previously observed TGS due to homology in promoter regions and other studies correlated it with increased promoter methylation. Mette et al. (2000) showed that both TGS and PTGS can be triggered by dsRNA that enter the same degradation pathway in *Arabidopsis* and tobacco even though they occur in different cellular compartments. Several studies also reported that trans‐inactivation of transgenes can occur due to homology found in the promoter region and 3' region in the stacking of transgenes in a single transgenic line (Fagard & Vaucheret, 2000; Vaucheret, 1993, 1994). Our data also suggested that gene silencing was able to be induced *in‐trans* (i.e. from separate plasmids) – co‐infiltration of a DsRed plasmid and a 4 TU plasmid gave the same level of DsRed expression as when the DsRed TU was part of a 5 TU plasmid (Figure 2b), suggesting the level of silencing was similar in both cases. With the caveat that these were transient expression experiments, this result might have implications for multi‐gene expression by sexual crossing in stably transformed plants to achieve gene stacking, where expression of each transgene is driven by the same promoter.

To circumvent silencing due to high transgene dosage and/or exogenous promoter direct transgene silencing, the presence of repeated sequences could be reduced by employing endogenous plant promoters such as PD1 (Jiang et al., 2018) and ubiquitin‐10 (Grefen et al., 2010) in some of the TUs. Using different genetic elements such as non‐competing vectors (Teh et al., 2021) or 5ʹ and 3ʹ UTRs (Diamos & Mason, 2018), have also been shown to increase protein expression levels, even when the same genetic elements were used in multiple TUs (Diamos et al., 2020). Furthermore, BeYVD genetic elements (Diamos et al., 2020) can be used to “divide” transient expression vectors of more than 3 TUs into smaller operons when the vector is processed in the nucleus.

Interestingly, when DsRed was expressed as the first or last TU in a 5 TU construct, expression was restored to levels comparable to when it was expressed as part of a 3 TU construct. This might also be due to the stability of the DsRed transcript. When expressed as the first or last TU in a 5 TU construct, mRNA transcripts were detected at levels lower than when VRC01 HC was expressed as the last TU. However, DsRed expression was detected while VRC01 HC was not. This demonstrated that the level of mRNA expression might be sufficient for the expression of detectable levels of DsRed. It would be interesting to quantify the abundance and stability of DsRed mRNA transcripts using a noninvasive mRNA labeling method such as thio‐modified uracil (Chan et al., 2018), and correlate it with the expression levels of DsRed protein in plants expressing permutations of the 5 TU construct.

In summary, we have shown that MIDAS‐P, with its capacity to iteratively add TUs based on alternating screening of white and blue colonies, is a highly practical and predictable cloning method for the assembly of multiple genes in tandem in one vector. Using the MIDAS‐P constructs, we successfully expressed an anti‐HIV protein cocktail expressing the broadly neutralizing antibody VRC01 and the lectin GRFT, with expression levels reaching up to approximately 0.8 g/kg for VRC01 and approximately 5 g/kg for GRFT with co‐expression of the silencing suppressor P19. This study has highlighted the limitations associated with repeated use of the same sequence elements (promoter, terminator, SAR) and future studies will aim to further extend the repertoire of TU modules optimized for plant expression to improve the number of genes that can be expressed transiently in tandem; and to mitigate the gene silencing effects observed upon coexpression of high numbers of transgenes.

## CONFLICTS OF INTEREST

The authors declare no conflicts of interest.

## AUTHOR CONTRIBUTIONS

Craig J. van Dolleweerd, Audrey Y‐H. Teh, and Julian K‐C. Ma provided substantial contributions to the conception of the work. All authors substantially contributed to the acquisition, analysis, or interpretation of data for the manuscript and drafting, revising, and critically reviewing the manuscript for important intellectual content.

## Supporting information

Supporting information.

## Data Availability

The data that support the findings of this study are available from the corresponding author upon reasonable request.
