# Supplementary material for: Multiple gene expression in plants using MIDAS‐P, a versatile type II restriction‐based modular expression vector
Source: Biotechnol Bioeng. 2022 Mar 16;119(6):1660–72. doi: 10.1002/bit.28073 (PMC9313558; doi:10.1002/bit.28073)
Supplement: Supplementary file 1 — Supporting information. [file BIT-119-1660-s001.docx]

**Supplementary Table S1:** Primers used during construction of MIDAS-P constructs and cloning genes of interest. SAR – Scaffold Attachment Region. CAMV 35S term – Cauliflower Mosaic Virus 35S terminator.

|  | **Forward primer** | **Reverse Primer** | **Template** |
| --- | --- | --- | --- |
| AarI[GGAC]-BsaI[CATT]-SAR-BstBI-BamHI | 5’ TCCGCTCACCTGCCTCAGGACGGTCTCCCATTGAAGAG 3’  5’ GGACGGTCTCCCATTGAAGAGAAAGCTAATTCCGCC 3’ | 5’ TATCGAGGATCCACTTCAAATTCGAAAAAGATATATAAG 3’ | pTRAK-2G12LC |
| BamHI-XbaI-CAMV 35S term-[CATT]BsmBI-BsmBI[CGTA]BsaI-[GCT]SapI | 5’GGAGAGGGATCCTCTAGAGTCCGCAAAAATCACCAG 3’ | 5’ CGCAAGCTAAACGTCTCCAATGGCCCGGTCACTGGATTTTGGTTT 3’  5’ TCTCTTACGTGAGACGGCTGACGCAAGCTAAACGTCTCCAATG 3’  5’ GTGGTTGCTCTTCGAGCGGGTCTCTTACGTGAGACGGCTGAC 3’ | pTRAK-2G12LC |

**Supplementary Table S1 (continued):** Primers used during construction of MIDAS-P constructs and cloning genes of interest. SAR – Scaffold Attachment Region. CAMV 35S term – Cauliflower Mosaic Virus 35S terminator.

|  | **Forward primer** | **Reverse Primer** | **Template** |
| --- | --- | --- | --- |
| AarI[GGAC]-BsmBI[CATT]-SAR-BstBI-BamHI | 5’ TCCGCTCACCTGCCTCAGGACCGTCTCCCATTGAAGAG 3’  5’ GGACCGTCTCCCATTGAAGAGAAAGCTAATTCCGCC-3’ | 5’ TATCGAGGATCCACTTCAAATTCGAAAAAGATATATAAG 3’ | pTRAK-2G12LC |
| BamHI-XbaI-CaMV 35S term-[CATT]BsaI-BsaI[CGTA]BsmBI-[GCT]SapI | 5’ GGAGAGGGATCCTCTAGAGTCCGCAAAAATCACCAG 3’ | 5’ CGCAAGCTAAAGGTCTCCAATGGCCCGGTCACTGGATTTTGGTTT-3  5’ TCTCTTACGTGAGACCGCTGACGCAAGCTAAAGGTCTCCAATG-3’  5’ GTGGTTGCTCTTCGAGCGCGTCTCTTACGTGAGACCGCTGAC-3’ | pTRAK-2G12LC |
| SapI[CAT]-[CATT]BsaI-*lac* promoter-*lacZα*-BsaI[CGTA]FseI | 5’ TCAAGGGCTCTTCACATTG  GAGACCGCGCAACGCAATTAATGTG 3’ | 5’ CCTGCAGGCCGGCCTACGCGAGACCTCACCATTCGCCATTCAGGCTG 3’ | pNUTKan01 |
|  |  |  |  |

**Supplementary Table S1 (continued):** Primers used during construction of MIDAS-P constructs and cloning genes of interest. SAR – Scaffold Attachment Region. CAMV 35S term – Cauliflower Mosaic Virus 35S terminator.

|  | **Forward primer** | **Reverse Primer** | **Template** |
| --- | --- | --- | --- |
| VRC01 HC | 5’ GCGCCCATGGCTAACAAGCACCT 3’ | 5’ GCGCTCTAGACTACTTACCAGGAGACAGAGAC 3’ | pTRAk.2 VRC01 HC+LC (Teh *et al.*, 2014) |
| VRC01 LC | 5’ GCGCCCATGGCTAACAAGCACCT 3’ | 5’ GCGCTCTAGACTAGCACTCTCCCCTATTAAAAG 3’ | pTRAk.2 VRC01 HC+LC (Teh *et al.*, 2014) |
| GRFT | 5’ ATCCATGGCTTCTCTTACTCATAGAAAG 3’ | 5’ ACTCTAGAGTCAGTACTGCTCGTAGTA 3’ | pHK20-GRFT  (Hoelscher *et al.*, 2018) |
| J3-VHH | 5’ATCCATGGAGGTGCAGCT 3’ | 5’ TGCTCTAGACTAGTGATGGTGATGGTGATGGCCTGAGAAGAC 3’ | pCAD51-J3VHH (McCoy *et al.*, 2012) |
| DsRed | 5’ TACGAAGGTCTCCCATGGCGGCTCTGGCCA 3’ | 5’ CGGACTGGTCTCTCTAGACTAAAGGAACAGATGGTGGCCGTCC 3’ | pTRAp-2G12-DsRed  (Ma *et al.*, 2015) |
| P19 | 5’ ATCCATGGAACGAGCTATACAAGGA 3’ | 5’ ACCTCTAGATTACTCGCTTTCTTTTTCGAA 3’ | pEAQ-HT-DEST3 (Sainsbury *et al.*, 2009) |

**Supplementary Table S2:** Primers used for Taqman qPCR.

| **Gene** | **Forward primer** | **Reverse primer** | **Internal oligo probe** |
| --- | --- | --- | --- |
| VRC01 HC | 5’ AGTTATTGTGTCATCTGCTTCTACTAAG 3’ | 5’ AAGAAAGAGAGTAAAGACCAGAAGATT 3’ | 5’ ATCTAAGTCTACCTCTGGTGGTACTGCT 3’ |
| VRC01 LC | 5’ TGGTGAAACCGCTATTATTT 3’ | 5’ TTAGTACCCTGACCGAAAAA 3’ | 5’ TTTTCTGGTTCAAGATGGGG 3’ |
| GRFT | 5’ GTTCTTACCTGGATGCTATTATCAT 3’ | 5’ GAAGCTGATGTTATCGATGTAATCA 3’ | 5’ GGTGTGCATCATGGTGGATCTGGTGGT 3’ |
| J3His | 5’ ACTCTCCTGTGAACTTCGTG 3’ | 5’ GGTTTCAGGCTACTCATTTG 3’ | 5’ GGAAGGAGCGTGAGTTTGTC 3’ |
| DsRed | 5’ AAGGATTTAAATGGGAAAGG 3’ | 5’ GAATCTCTCCTTTCAACACG 3’ | 5’ ACTTTCCTTCCGATGGACCT 3’ |
| L25 | 5’ TTTGAATTTTGCGTTTCAGA 3’ | 5’ AACACATTTGTCTCCCGAAT 3’ | 5’ TCCTTAGGCAATTCTGCTACCC 3’ |

**
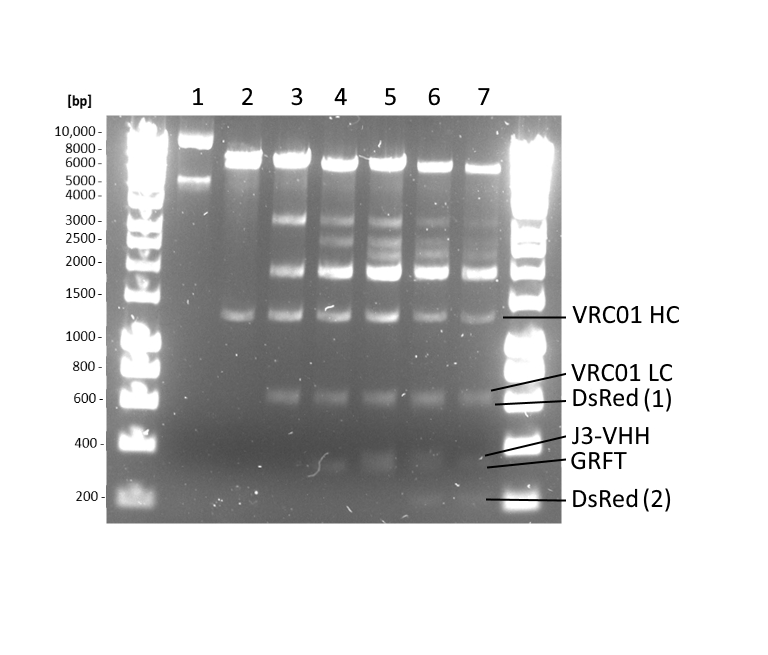
**

**Supplementary Figure S1:** Restriction analysis of plasmids purified from *Agrobacterium*. Plasmids were isolated from *Agrobacterium* using a plasmid extraction kit (Qiagen, USA) per manufacturer’s instructions and propagated in *Escherichia coli* DH10B**.** Plasmids isolated from DH10B were analysed by restriction digestion with NcoI and XbaI followed by electrophoresis on a 1% (w/v) agarose gel. pMIDAS only (Lane 1), pMIDAS-VRC01 HC (Lane 2), pMIDAS-VRC01 HC-VRC01 LC (Lane 3), pMIDAS-VRC01 HC-VRC01 LC-GRFT (Lane 4), pMIDAS-VRC01 HC-VRC01 LC-GRFT-J3VHH (Lane 5), pMIDAS-VRC01 HC-VRC01 LC-GRFT-J3VHH-DsRed (Lane 6) and pMIDAS-DsRed-J3VHH-GRFT-VRC01 LC-VRC01 HC (Lane 7). Hyperladder 1kb DNA ladder (Meridian Bioscience, USA) was used as size markers. Restriction digest with NcoI and XbaI showed that the plasmids were intact in *Agrobacterium* with no recombination-mediated rearrangements or deletions. DNA fragments showed VRC01 heavy chain (~1400bp) and light chain (~700bp), as well as DsRed (1)(2) (~680bp/~220bp), J3-VHH (~400bp) and Griffithsin (~380bp). Fragments between ~2000 and ~3000bp were fragments in between the genes which included promoter, enhancer and terminator elements as well as the *lacZ* expression cassette. Gel is representative of 2 biological repeats.

**
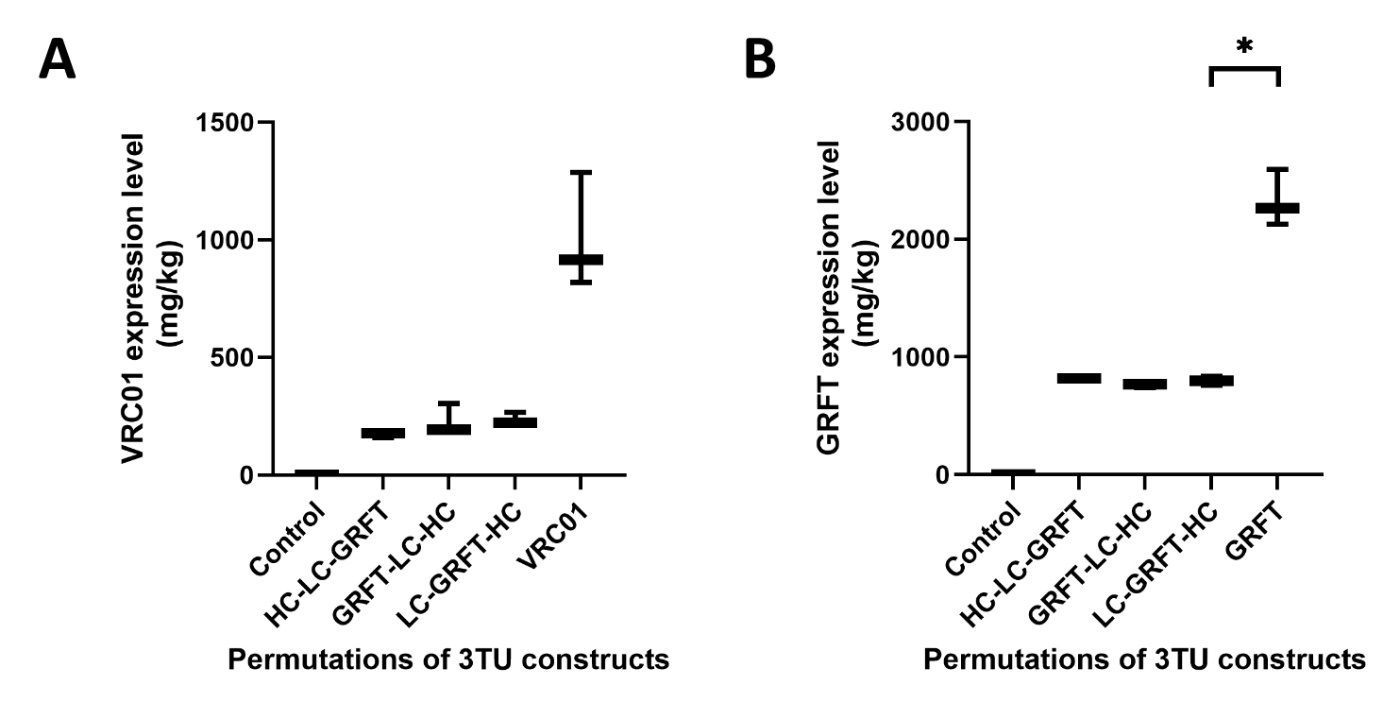
**

**Supplementary Figure S2:** VRC01 or Griffithsin (GRFT) expression levels in permutations of 3 TU constructs at 6 days post-infiltration. VRC01 (A) and GRFT (B) expression levels were quantified by ELISA using a human IgGk standard (Sigma-Aldrich, USA) and GRFT standard (gift from Paul Christou) respectively. Mock infiltration containing only pMIDAS was used as negative control (Control). Box plots for VRC01 and GRFT expression levels represent the mean, minimum and maximum of three biological repeats. Data were analysed using Brown-Forsythe and Welch ANOVA tests with Tamhane T2 multiple comparison test (*p<0.033).

**
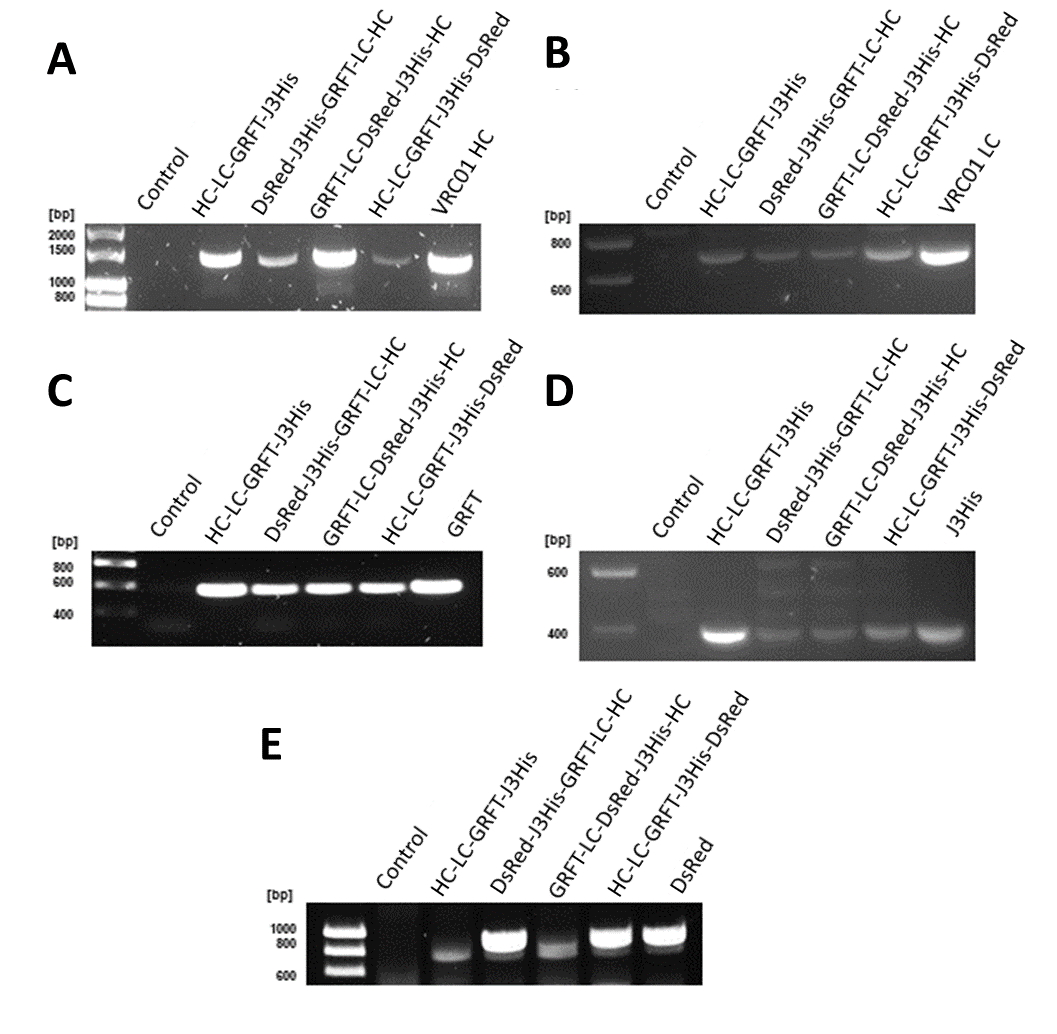
**

**Supplementary Figure S3:** PCR amplification of targets from genomic DNA isolated from 6-day old *N. benthamiana* leaves infiltrated with 4 and 5 TU constructs compared to positive controls infiltrated with 1 TU (for GRFT, J3His and DsRed) or 2 TUs (for VRC01), confirming the presence of VRC01 heavy chain (A), VRC01 light chain (B), Griffithsin (C), Histidine tagged J3-VHH (D), and DsRed (E). Leaves infiltrated with pMIDAS only was used as a negative control (Control). HC-LC-GRFT-J3His was shown as a representative example of 4 TU construct.

**
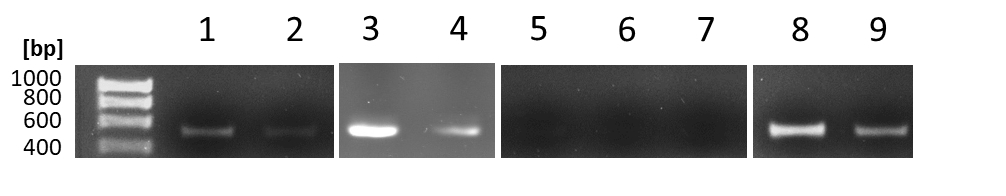
**

**Supplementary Figure S4:** Verification of *p19* by RT-PCR. mRNA was extracted from *N. benthamiana* infiltrated with pMIDAS-P19 (1), pMIDAS-P19 with pMIDAS-VRC01 HC&LC (2), pMIDAS-P19-VRC01 HC-LC (3), pMIDAS-VRC01 HC-LC-P19 (4), pMIDAS-VRC01 HC&LC only (5), pMIDAS only (6), and PBS only (7). cDNA was made from mRNA extracted from infiltrated leaves and TBSV *p19* gene was amplified with P19 forward and reverse primers. The primers were also used in a PCR reaction of 50ng and 10ng of pMIDAS-P19 construct were used as positive control (8 and 9 respectively). PCR products ran on a 1% Agarose gel with a Hyperladder 1kb DNA ladder (Meridian Bioscience, USA) showed bands at ~500bp, consistent with the size of P19. Figure representative of 2 repeats.

**
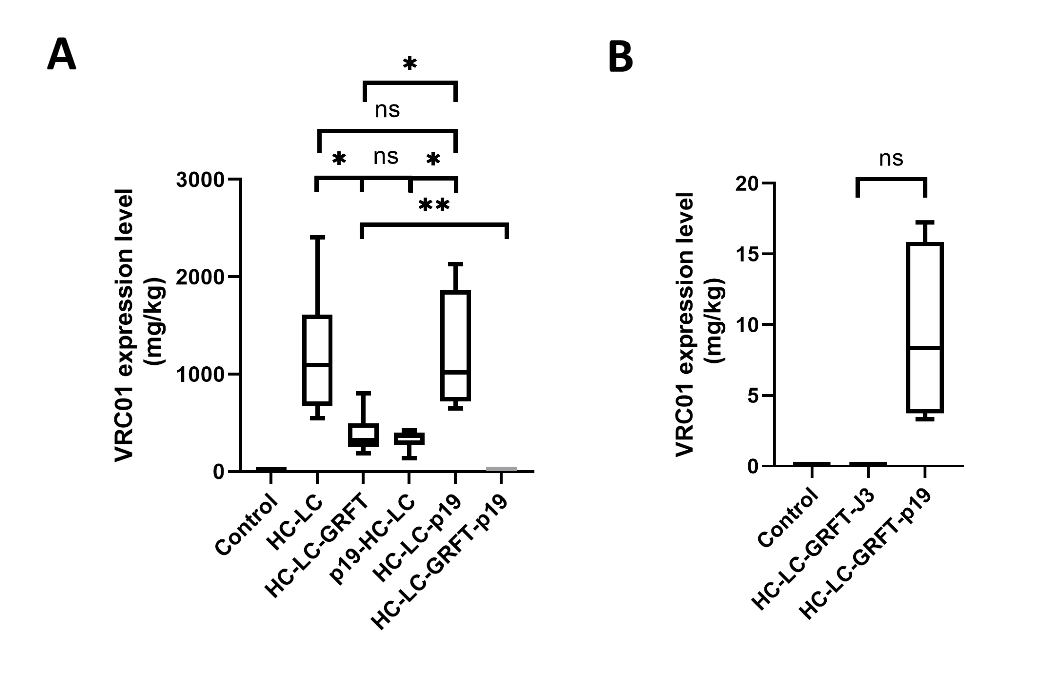
**

**Supplementary Figure S5:** VRC01 expression levels when P19 was co-expressed in the same construct. HC+LC (VRC01 heavy and light chain), HC-LC-GRFT and HC-LC-GRFT-J3 constructs were used as positive controls. Mock infiltration containing only pMIDAS was used as negative control (Control). VRC01 expression levels were quantified by ELISA using a human IgGk standard (Sigma-Aldrich, USA). Box plots for VRC01 expression levels represent the mean, minimum and maximum of six biological repeats. Data were analysed using Brown-Forsythe and Welch ANOVA tests with Tamhane T2 multiple comparison test (*p<0.033, **p<0.05).
